# UHPLC-Q/TOF-MS-based differential metabolite screening and origins classification of Codonopsis Radix

**DOI:** 10.3389/fphar.2026.1751079

**Published:** 2026-02-18

**Authors:** Sihan Xuchen, Yong Zhang, Shiyuan Fang, Huizhen Li, Yue Ding, Tong Zhang

**Affiliations:** 1 School of Pharmacy, Shanghai University of Traditional Chinese Medicine, Shanghai, China; 2 National Innovation Platform for Medical Industry-Education Integration, Shanghai University of Traditional Chinese Medicine, Shanghai, China

**Keywords:** Codonopsis Radix, neural network model, ratio method, UHPLC-Q/TOF-MS, untargeted metabolomics

## Abstract

**Introduction:**

Codonopsis Radix (CR), also known as Dangshen, is a renowned plant native to China, highly valued for its unique medicinal properties. However, due to the existence of numerous closely related origins and the similarities in their interspecies traits, microscopic characteristics, and physicochemical properties, different origins of CR have been circulating in the market, posing significant challenges for standardizing its medicinal use. Therefore, establishing an accurate identification method is crucial for advancing research on CR and ensuring its proper utilization.

**Methods:**

In this study, a UHPLC-Q/TOF-MS analysis method was developed to identify differential metabolites among three origins of CR using one-way analysis of variance (ANOVA). The metabolites were distinguished based on the response ratios of the differential metabolites. Additionally, a neural network (NN) model was established to validate the classification capability.

**Results:**

Metabolomic results revealed that among the 56 identified metabolites, 29 differential metabolites were screened out. Notably, the response ratios of codonopyrrolidium A, codonopyrrolidium D, tryptophan, and codonopsinol A against 3′-hydroxy codonopyrrolidium B exhibited significant differences among the three origins. Verification experiments demonstrated that the NN model achieved a prediction accuracy of 100%, with a confidence measure exceeding 0.98.

**Discussion:**

This study established two methods for identifying the origins of CR: a simple and rapid ratio method, and a highly accurate NN model. It demonstrated the feasibility of identifying the origins of CR through cross-validation, providing new insights and methodologies for the origin identification of multi-origin traditional Chinese medicine.

## 1 Introduction

Codonopsis Radix (CR) is a commonly used traditional Chinese medicinal herb with both medicinal and culinary applications. It is traditionally applied to alleviate symptoms associated with spleen and lung deficiency. In Asian nations such as China, Japan, and South Korea, CR is highly valued and widely used as a key ingredient in various foods, including tea, wine, soup, porridge, and other dishes. In China, nearly 200 health food products containing CR have been approved (Zou et al., 2020). According to phytochemical studies, the primary compounds of CR are polysaccharides, polyacetylene, polyenes, lignans, alkaloids, nitrogen compounds, and terpenoids (Gao et al., 2018). Pharmacological studies have shown that CR can decrease blood glucose and blood lipids (Liu et al., 2018), improve liver function (Ma et al., 2024), regulate the immune system (Yin et al., 2019), slow down the aging process of the brain (Wang et al., 2024) and prevent the beginning of Alzheimer’s disease (Zhao et al., 2024). Thus, China uses 40,000 tons of CR annually, which is a significant amount of consumption and holds great economic importance (Gao et al., 2018).

Within the Campanulaceae family, CR is a dicotyledonous herbaceous perennial plant with over 40 species, 39 of which are found in China alone (He et al., 2015). *Codonopsis pilosula* (Franch.) Nannf. (CP), *C*. *pilosula* Nannf.var.*modesta* (Nannf.) L.T.Shen (CM) and *C*. *tangshen* Oliv. (CT) are widely used and recognized by the Chinese Pharmacopoeia (2025) due to their excellent quality (Commission Pharmacopoeia Commission, 2025). Because of the high market demand and the depletion of wild resources, CP, CM, and CT are extensively cultivated (Bai et al., 2021a). CR from Gansu Province is widely recognized for its superior efficacy. Both CP and CM from Gansu have been approved as Geographical Indication products by Chinese authorities. Consequently, CR collected in Gansu Province generally commands a higher market price compared to that from other provinces, with CM being particularly prized and often more expensive than other varieties (Bai et al., 2021b). There may be unscrupulous businessmen confusing three kinds of CR and disrupting the medicinal materials market. The inability to distinguish among the three origins poses a challenge for both growers and consumers. More importantly, research has indicated that metabolite content varies among different original CR (Li et al., 2023), leading to differences in efficacy. Therefore, it is crucial to develop a rapid and reliable method for analyzing the differences in metabolites among CM, CP, and CT, and for accurately distinguishing their origins.

Morphological and microscopic identification are widely used for CR authentication but suffer from subjective interpretation, limiting their reproducibility (Wang et al., 2022). Differentiation becomes particularly challenging when CR is processed into slices, as showed in Supplementary Figure S1. The Chinese Pharmacopoeia (2025) relies solely on lobetyolin for thin-layer chromatography, which lacks specificity for origin discrimination. Although metal element analysis (Bai et al., 2021b) and HPLC-ELSD oligosaccharide fingerprinting with chemometrics (Bai et al., 2022) can distinguish CR origins, the former is prone to environmental interference, while the latter involves cumbersome preprocessing. These limitations underscore the urgent need for a rapid, simple, and accurate origin-tracing methodology for CR. In recent years, the research of medicinal and edible medicinal materials has made use of the appealing method known as MS-based analysis, which has been utilized for the identification, screening, and systematic characterization of possible bioactive compounds and chemical markers (Cavanna et al., 2018). For instance, untargeted metabolomics using UHPLC-Q/TOF was employed to identify licorice metabolites. Subsequently, chemometrics was utilized to assess the metabolomics data and develop a geographical differentiation model for licorice (Lu et al., 2022). The metabolic profiling based on LC-ESI-Q/TOF-MS, combined with chemometric analysis, can be utilized to identify the primary and specialized metabolites responsible for discrimination, thereby distinguishing *Butia* species and the geographical origins of *Butia odorata* (Hoffmann et al., 2017).

Therefore, this study established a robust UHPLC-Q/TOF-MS analytical method to identify metabolites in CR. Subsequently, chemometric approaches were employed to evaluate metabolomics data and screen differential metabolites among CR origins. Finally, a dual-strategy framework integrating a ratio-based method and a neural network (NN) model was developed using these screened biomarkers, enabling rapid discrimination of CR origins. The proposed methodology provides novel insights and practical approaches for the rapid classification of other multi-origin medicinal materials.

## 2 Materials and methods

### 2.1 Sample collection

A total of 38 batches of CR were collected in this study, of which 13 batches were CP, 16 batches were CM, and nine batches were CT. All samples were botanically authenticated by Prof. Yue Ding. The key morphological identification characteristics of three CR origins are listed in Table 1. The batch number and origin information about the samples are presented in Supplementary Table S1.

**TABLE 1:** Key morphological identification characteristics for the authentication of three CR origins.

Morphological characteristic	CP	CM	CT
Size	10–35 cm long, 0.4–2 cm in diameter	10–35 cm long, 0.5–2.5 cm in diameter	10–45 cm long, 0.5–2 cm in diameter
Surface	Grayish-yellow to yellowish-brown; densely covered with annular transverse wrinkles below the root head, becoming sparse downward	Yellowish-white to grayish-yellow; dense annular transverse wrinkles often extend over half of the length	Grayish-yellow to yellowish-brown; exhibits distinct irregular longitudinal furrows
Root head	With numerous wart-like stem scars and buds, each scar with a concave, dot-like apex	Similar to CP.	Similar to CP.
Texture	Slightly soft or hard yet tenacious	Similar to CP.	Softer yet compact
Fracture surface	Relatively even; exhibits fissures or radial striations; bark pale brownish-yellow to yellowish-brown, wood pale yellow to yellow	More fissured; bark grayish-white to pale brown	Fewer fissures; bark yellowish-white
Odor and taste	Characteristic aromatic odor; slightly sweet taste	Characteristic aromatic odor; slightly sweet taste	Characteristic aromatic odor; slightly sweet taste

### 2.2 Reagents

Reference compounds including adenosine, tryptophan, tangshenoside I, coniferoside, lobetyolinin, atractylenolide I, atractylenolide II, atractylenolide III, and (E)-isoconiferin were obtained from Shanghai Hongyong Bio-Technology Co. Ltd. (Shanghai, China); lobetyolin and syringin were obtained from National Institutes for Food and Drug Control (Beijing, China); nicotinic acid, maleic acid, caffeic acid and chlorogenic acid were obtained from Shanghai yuanye Bio-Technology Co. Ltd. (Shanghai, China). The reference compounds had purities >98%. LC-MS-grade methanol and acetonitrile were acquired from Merck (Darmstadt, Germany). LC-MS-grade formic acid (purity >98%) was obtained from Tokyo Chemical Industry Co. Ltd. (Tokyo, Japan). Ultrapure water was sourced from a local market (Watsons, China).

### 2.3 Metabolite extraction

In this study, an extraction method was established to pretreat all CR. CR was crushed using a powderer and passed through a No. 3 sieve (50 mesh). Extraction was performed under optimized conditions. 1.5 g of the powder was weighed, added with 25 mL 70% methanol solution (v/v) and ultrasonicated at 25 °C for 30 min (53 kHz). The extract was then centrifuged at 15,000 rpm for 15 min. Finally, the supernatant was collected for UHPLC-Q/TOF-MS analysis. All samples were processed three times in parallel.

All samples were mixed into quality control (QC) samples in equal volumes (1 mL for each). The QC sample was analyzed five times initially before the start of the sequence to ensure the equilibration of the analysis system. Additionally, to maintain the system stability, the QC sample was analyzed after every five CR samples (Tao et al., 2018). The relative standard deviations (RSDs) of the peak area and retention time, along with the mass difference—defined as the difference between the observed and calculated mass—of the selected ions (*m/z* 268.1543, 350.1962, and 231.1380) in all QC samples were calculated to assess the reproducibility and stability of the analytical method.

### 2.4 UHPLC-Q/TOF-MS analysis

A UHPLC-Q/TOF-MS method was established. Samples were analyzed on an Agilent 1290-6545 UHPLC-Q/TOF-MS system. An Agilent ZORBAX Eclipse Plus C18 column (100 mm × 2.1 mm, 1.8 μm) was used for chromatographic separation. The mobile phase consisted of 0.1% formic acid in water (A) and acetonitrile (B). A gradient elution (0 ∼ 15 min, 5% ∼ 20%B; 15 ∼ 20 min, 20% ∼ 40%B; 20 ∼ 25 min, 40% ∼ 60%B; 25 ∼ 35 min, 60% ∼ 90%B; 35 ∼ 40 min, 90% ∼ 90%B) was optimized. The mobile phase was maintained at a flow rate of 0.4 mL/min, with an injection volume of 2 μL and a column temperature of 30 °C. Ionization was achieved using an electrospray ionization source (ESI). The Q/TOF parameters were set as follows: *m/z* scan range of 100–2,000 for MS and 50–2,000 for MS/MS, with acquisition rates of 2 spectra/sec for MS and 4 spectra/sec for MS/MS; collision energies of 10 eV and 30 eV; sheath gas temperature at 350 °C; sheath gas flow at 11 L/min; dry gas flow at 8 L/min; nebulizer pressure at 35 psi; Vcap set to 3,500 V.

### 2.5 Metabolite identification

The original data were processed by Agilent software Qualitative Analysis 10.0, and both the primary and secondary mass spectrometry data were qualitatively analyzed based on the self-built CR compound database of the Agilent software Personal Compound Database Library Manager B.08.00. The database was established based on the public database of metabolite information (https://pubchem.ncbi.nlm.nih.gov/; https://old.tcmsp-e.com/tcmsp.php) and literature (Gao et al., 2019; Jia et al., 2022; Ma et al., 2014; Tang et al., 2022; Yue et al., 2023). Some compounds were also identified with the help of commercial standards analyzed under the same chromatographic conditions.

### 2.6 Statistical analysis

Raw data were preprocessed using Agilent MassHunter Profinder 10.0 software. The minimum peak height was 1,000, and the RT tolerance and masses tolerance were 0.20 min and 10 ppm, respectively. The processed data were subjected to non-targeted metabolomics analysis by Agilent Mass Profiler Professional (MPP) software. To make sure the data were comparable, all the data were normalized at 75% percentile to remove redundant data. The first filter was frequency analysis, which was set to 25% to ensure 25% of compounds present in at least one studied group (Amir Hashim et al., 2021). The significant differential metabolites of the samples were screened by one-way analysis of variance (ANOVA). The p-value was <0.05, and the fold change value was ≥2. The peak area of the significantly different metabolites was log transformed, and the classification ability of the different compounds was assessed by data analysis. The classification models of different origins of CR were established by using differential metabolites, and the classification accuracy of different models was compared. Then, the verification experiment was carried out on the model with the best classification performance, which proved that it had the excellent ability to correctly classify different origins of CR.

## 3 Results and discussion

### 3.1 Method validation

**FIGURE 1:**
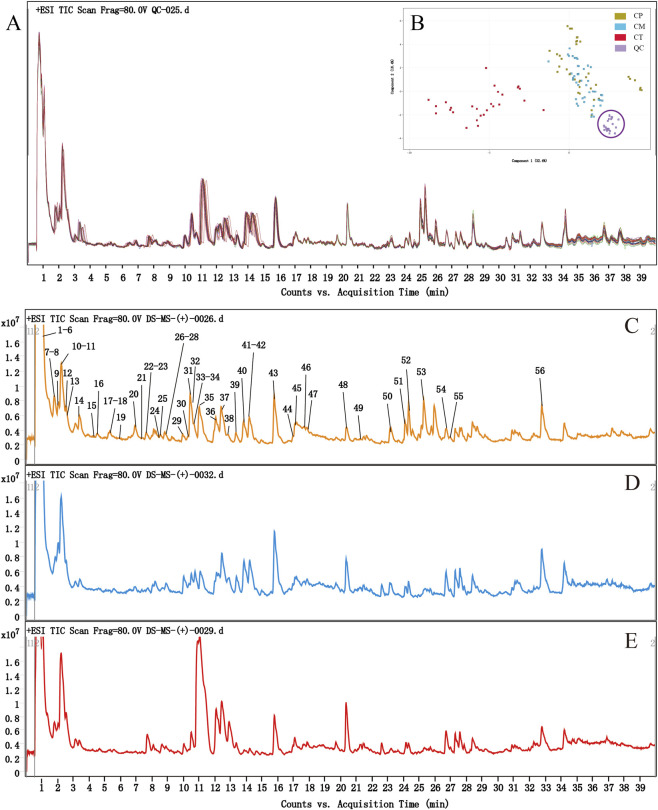
TIC overlapping map of QC samples mass spectrometry results **(A)**; PCA of CP, CM, CT and QC samples **(B)**; TIC compounds identification diagram of CP in ESI+ **(C)**; TIC of CM **(D)** and CT **(E)** in ESI+.

To maximize metabolite detection, we optimized a widely-used extraction procedure for our specific samples. This enabled the development of a tailored UHPLC-Q/TOF-MS method that is both stable and reliable, successfully maximizing the number of resolved metabolite peaks within a practical runtime while maintaining high separation quality. To validate the UHPLC-Q/TOF-MS method, we calculated the mass difference and the RSDs of the peak area and retention time for the selected positive ions with retention time of 2.23 min (*m/z* 268.1543), 11.07 min (*m/z* 350.1962), and 27.03 min (*m/z* 231.1380) in all QC samples (n = 25). The data indicate that the mass difference of the selected ions was less than 10 ppm. The RSDs of retention time of all selected ions were below 1%, while the RSDs for peak areas were 3.99% (*m/z* 268.1543), 2.63% (*m/z* 350.1962) and 6.30% (*m/z* 231.1380), respectively. Figure 1A shows the overlapping display analysis of the total ion current (TIC) results from various QC samples. The high overlap of TIC curves from different QC samples indicates that the evaluation results are repeatable and reliable. Additionally, system equilibration was further evaluated by examining the distribution of QC samples in the principal component analysis (PCA) plot. As shown in Figure 1B, the QC samples are closely clustered around the center, indicating high repeatability and reliability of the results (Xiao et al., 2022). These results confirmed that the entire analysis process had good stability and the data recorded in this study had high repeatability and reliability.

### 3.2 Metabolite identification

In this study, the metabolites in CR samples were analyzed by advanced UHPLC-Q/TOF-MS analysis technology. The TIC (TIC+) of different original CR in the positive ionization mode (ESI+) is shown in Figures 1C–E, while the TIC (TIC−) in the negative ionization mode (ESI−) is shown in Supplementary Figure S2. From the results, there is no significant difference in the composition of CP, CM and CT, but the content of some metabolites is significantly different. The amount of information expressed by TIC− is limited, with no significant differences between CP, CM and CT. TIC+ expresses more information, and it can be seen that the intensity of many peaks is significantly different between CP, CM and CT.

Q/TOF-MS and Q/TOF-MS/MS can provide precise molecular weights and detailed chemical structural characteristics of metabolites (Shi et al., 2022). A total of 56 metabolites were identified based on fragment ions, reference mass spectral data, and literature sources. These include 18 alkaloids, 16 organic acids, 7 lignans, 3 polyacetylenes, 4 terpenoids and 8 other types of compounds. The mass information is listed in Table 2, including retention time, adduct ion, detected *m/z*, calculated *m/z*, error value, molecular formula, MS/MS data of identified metabolites and type. Among them, 15 metabolites have been compared with reference substances. Taking polyacetylenes and alkaloids as examples, the process of compound identification was illustrated.

**TABLE 2:** Qualitative analysis of CR using UHPLC-Q/TOF-MS.

No	RT (min)	Adduct ion	Detected *m/z*	Calculated *m/z*	Error (ppm)	Formula	Compounds	MS/MS	Type
1*	0.796	[M − H]−	115.0045	115.0037	6.84	C_4_H_4_O_4_	Maleic acid	71.0137, 59.0136	Org
2*	0.896	[M + H]+	124.0396	124.0393	2.54	C_6_H_5_NO_2_	Nicotinic acid	96.0436, 80.0497, 69.0334, 53.0388	Org
3*	1.006	[M + H]+	268.1047	268.1040	−0.07	C_10_H_13_N_5_O_4_	Adenosine	136.0622, 119.0350, 74.0599, 57.0334	Alk
4	1.053	[M + H]+	270.1343	270.1336	1.97	C_13_H_19_NO_5_	Codonopsinol A	177.0543, 74.0605	Alk
5	1.088	[M]+	284.1503	284.1492	3.70	C_14_H_22_NO_5_^+^	3′-hydroxy codonopyrrolidium B	177.0545, 152.0566, 118.0861, 88.0764	Alk
6	1.096	[M − H]−	117.0196	117.0193	2.20	C_4_H_6_O_4_	Succinic acid	99.0080, 73.0297	Org
7	1.885	[M + H]+	416.1917	416.1915	0.05	C_19_H_29_NO_9_	Codonopiloside A	254.1382, 205.0855, 161.0612, 74.0603	Alk
8	1.992	[M + H]+	127.0388	127.0390	−1.35	C_6_H_6_O_3_	5-Hydroxymethyl-2-furaldehyde	109.0285, 81.0343, 53.0929	Ter
9	2.068	[M + H]+	254.1393	254.1387	1.97	C_13_H_19_NO_4_	Codonopsinol B	205.0849, 161.0597, 74.0602	Alk
10	2.234	[M]+	268.1551	268.1543	2.85	C_14_H_22_NO_4_^+^	Codonopyrrolidium B	161.0595, 121.0645, 88.0761	Alk
11	2.327	[M − H]−	329.0882	329.0878	1.42	C_14_H_18_O_9_	Woodorien	271.1547, 167.0342	Oth
12	2.550	[M + H]+	268.1547	268.1543	1.40	C_14_H_21_NO_4_	Codonopsine	161.0595, 98.0601, 88.0766, 58.0657	Alk
13	2.560	[M − H]−	137.0244	137.0244	0.01	C_7_H_6_O_3_	4-Hydroxybenzoic acid	125.9976, 109.0290, 93.0346, 65.0040	Org
14*	3.399	[M + H]+	205.0977	205.0972	2.43	C_11_H_12_N_2_O_2_	Tryptophan	188.0709, 159.0916, 146.0600, 132.0804, 118.0652, 84.9596	Oth
15	4.374	[M − H]−	153.0194	153.0193	−1.94	C_7_H_6_O_4_	Protocatechuic acid	109.0289, 96.9595, 82.9547, 61.9872	Org
16*	4.531	[M + Na]+	365.1205	365.1207	−0.10	C_16_H_22_O_8_	Coniferoside	235.9555, 203.0679, 185.0395, 60.0801	Lig
17*	5.265	[M + H]+	355.1028	355.1024	0.74	C_16_H_18_O_9_	Chlorogenic acid	163.0389, 145.0280, 135.0437, 117.0333, 89.0388	Org
18*	5.380	[M + Na]+	395.1306	395.1313	−1.84	C_17_H_24_O_9_	Syringin	294.1274, 233.0788, 203.3965, 185.0440, 60.0500	Lig
19*	6.022	[M − H]−	179.0351	179.0350	0.66	C_9_H_8_O_4_	Caffeic acid	135.0452, 107.0498, 89.0241, 79.0545, 59.0141	Org
20*	7.024	[M + Na]+	365.1204	365.1207	−0.91	C_16_H_22_O_8_	(E)-Isoconiferin	349.0820, 303.2805, 203.0525, 185.0574, 60.0821	Lig
21	7.503	[M + COOH]−	417.1410	417.1402	1.58	C_17_H_24_O_9_	Tangshenoside II	371.1300, 209.8749, 179.0561	Lig
22	7.709	[M]+	366.1920	366.1911	2.42	C_19_H_28_NO_6+_	Codonopyrrolidium I	221.0801, 177.0545, 88.0758, 83.0495	Alk
23	7.759	[M + H]+	153.0546	153.0546	−0.54	C_8_H_8_O_3_	Vanillin	125.0599, 110.0371, 93.0330	Oth
24	8.591	[M]+	368.2067	368.2068	−0.17	C_19_H_30_NO_6_^+^	3′-hydroxy codonopyrrolidium F	221.0796, 177.0543, 88.0751, 85.0257	Alk
25	8.675	[M + H]+	369.1175	369.1180	−1.28	C_17_H_20_O_9_	3-*O*-caffeoylquinic acid methyl ester	177.0542, 145.0288, 134.0343, 117.0342	Org
26	8.991	[M]+	368.2078	368.2068	2.81	C_19_H_30_NO_6_^+^	3′-hydroxy codonopyrrolidium G	266.1379, 221.079, 177.0547, 88.0758, 85.0611	Alk
27	8.934	[M − H]−	325.0924	325.0929	−1.51	C_15_H_18_O_8_	p-coumaric acid glucoside	163.0402, 119.0501	Oth
28	9.085	[M + H]+	183.0653	183.0652	0.15	C_9_H_10_O_4_	Syringaldehyde	155.0695, 140.0648, 123.0439, 95.0493	Oth
29	10.053	[M + COOH]−	471.2080	471.2083	−0.74	C_18_H_34_O_11_	Hexyl-*β*-D-glucopyranosyl-(1→2)-*β*-D-glucopyranoside	425.2035, 263.1499, 101.0247	Oth
30	10.322	[M + H]+	195.0649	195.0652	−1.28	C_10_H_10_O_4_	Ferulic acid	177.0548, 145.0284, 117.0338, 89.0388	Org
31	10.389	[M]+	368.2078	368.2068	2.81	C_19_H_30_NO_6_^+^	3′-hydroxy codonopyrrolidium D	202.1421, 177.0563, 172.1330, 88.0747, 85.0630	Alk
32*	10.532	[M − H]−	677.2310	677.2298	1.71	C_29_H_42_O_18_	Tangshenoside I	497.1664, 453.1764, 261.0982, 161.0454	Lig
33	10.655	[M]+	368.2077	368.2068	2.54	C_19_H_30_NO_6_^+^	Codonopyrrolidium H	202.1433, 177.0545, 172.1331, 88.0754, 85.0643	Alk
34	10.796	[M + COOH]−	471.2079	471.2083	−0.97	C_18_H_34_O_11_	Hexyl-*β*-D-glucopyranosyl-(1→6)-*β*-D-glucopyranoside	425.2034, 263.1496, 161.0456, 101.0245	Oth
35	11.071	[M]+	350.1971	350.1962	2.57	C_19_H_28_NO_5_^+^	Codonopyrrolidium A	250.1438, 205.0858, 161.0599, 88.0761, 83.0495	Alk
36	12.103	[M]+	352.2129	352.2118	2.98	C_19_H_30_NO_5_^+^	Codonopyrrolidium F	250.1433, 220.1325, 205.0851, 161.0588, 88.0758, 85.1009	Alk
37	12.452	[M]+	352.2128	352.2118	2.70	C_19_H_30_NO_5_^+^	Codonopyrrolidium G	250.1435, 220.1332, 205.0861, 161.0598, 88.0762, 85.0651	Alk
38	12.918	[M]+	350.1970	350.1962	2.29	C_19_H_28_NO_5_^+^	Codonopyrrolidium C	250.1425, 170.1173, 88.0765, 83.0489	Alk
39*	13.401	[M + Na]+	581.2203	581.2205	−0.29	C_26_H_38_O_13_	Lobetyolinin	419.1664, 363.0883, 257.1152, 245.0621, 202.0447, 169.0482	Pol
40	13.850	[M]+	352.2129	352.2118	2.98	C_19_H_30_NO_5_^+^	Codonopyrrolidium D	172.1333, 161.0963, 88.0761, 85.0650	Alk
41	14.216	[M]+	352.2128	352.2118	2.70	C_19_H_30_NO_5_^+^	Codonopyrrolidium E	172.1336, 161.0955, 88.0762, 85.0652	Alk
42	14.243	[M − H]−	187.0980	187.0976	1.89	C_9_H_16_O_4_	Azelaic acid	169.0873, 125.0977, 97.0651	Org
43*	15.888	[M + Na]+	419.1665	419.1676	−2.87	C_20_H_28_O_8_	Lobetyolin	257.1160, 169.0477, 137.0559, 97.0242	Pol
44	16.989	[2M − H]−	699.3069	699.3081	−3.19	C_15_H_26_O_9_	Eucommioside II	349.1506, 305.1609	Oth
45	17.089	[M − H]−	823.2642	823.2666	−3.25	C_38_H_48_O_20_	Codonoside B	497.1663, 453.1744, 325.0937, 261.0982	Lig
46	17.638	[M − H]−	823.2642	823.2666	−3.00	C_38_H_48_O_20_	Codonoside A	497.1651, 453.1777, 325.0924, 261.0974, 99.0449	Lig
47	17.894	[M + Na]+	257.1142	257.1148	−2.49	C_14_H_18_O_3_	Lobetyol	169.0510, 143.0872, 103.0520, 91.0554	Pol
48	20.400	[M − H]−	329.2348	329.2333	3.96	C_18_H_34_O_5_	9,10,13-Trihydroxy-(E)-11-octadecenoic acid	229.1446, 211.1344	Org
49	21.249	[M − H]−	329.2339	329.2333	1.78	C_18_H_34_O_5_	5,6,9-Trihydroxy-octadec-7-enoic acid	229.1480, 211.1343	Org
50*	23.130	[M + H]+	249.1483	249.1485	−0.89	C_15_H_20_O_3_	Atractylenolide III	163.0747, 148.0833, 105.0691, 93.0700	Ter
51	24.112	[M-H]-	313.2402	313.2384	4.85	C_18_H_34_O_4_	Octadecenoic acid	295.2287, 277.2175, 183.1396, 99.0817, 58.0061	Org
52	24.345	[M − H]−	313.2399	313.2384	4.06	C_18_H_34_O_4_	9,10-Dyhydroxy-12-octadecenoic acid	295.2274, 277.2177, 201.1136, 127.1130	Org
53*	25.400	[M + H]+	233.1539	233.1536	1.05	C_15_H_20_O_2_	Atractylenolide II	215.1435, 187.1430, 151.0755, 131.0848, 105.0682, 81.0699	Ter
54	26.723	[M − H]−	295.2274	295.2279	−1.58	C_18_H_32_O_3_	9-Hydroxy-10,12-octadecadienoic acid	277.2177, 251.2406, 195.1391, 171.1025	Org
55*	27.031	[M + H]+	231.1376	231.1380	−1.43	C_15_H_18_O_2_	Atractylenolide I	203.1083, 185.1327, 157.1002, 143.0849, 129.0679, 105.0691, 81.0698	Ter
56	32.815	[M − H]−	279.2331	279.2330	0.52	C_18_H_32_O_2_	Linoleic acid	261.2188, 134.8933, 71.0140	Org

*indicates that the compound has been compared with the reference substance. Org, Alk, Ter, Lig, Pol and Oth indicate organic acids, alkaloids, terpenoids, lignans, polyacetylenes and other types of compounds, respectively.

**FIGURE 2:**
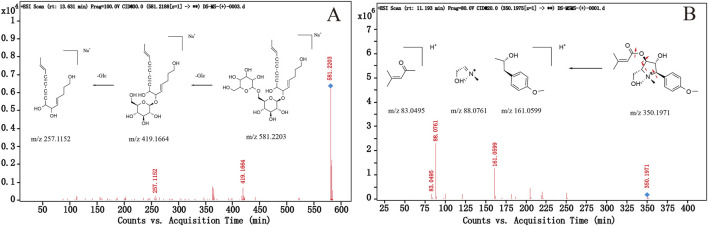
The proposed fragmentation pathway of lobetyolinin **(A)** and codonopyrrolidium A **(B)** based on its MS, MS/MS spectra, and reported data.

Polyacetylenes, lobetyol, lobetyolin and lobetyolinin are the main compounds of CR with antitumor, antioxidant, antiinflammatory, immunomodulatory activities (Xie et al., 2023). In the mass spectrometry of the identified polyacetylenes compounds, the formate anions [M + HCOO]^−^ were detected in the positive ionization mode, and the adduct ion of [M + H]^+^ and [M + Na]^+^ were produced in the positive ionization mode. Compound 43 (CP 43) produced *m/z* 441.1769 [M + COOH]^−^ adduct ions in the negative ionization mode and *m/z* 419.1678 [M + Na]^+^ adduct ions in the positive ionization mode. Compound 39 (CP 39) produced an adduct ion of *m/z* 581.2203 [M + Na]^+^ in the positive ionization mode. After losing a glycosyl group (162 Da), it produced a fragment ion of *m/z* 419.1664 [M + Na − Glc]^+^, and then losing a glycosyl group (162 Da) produced a fragment ion of *m/z* 257.1152 [M + Na − 2Glc]^+^. Compound 47 (CP 47) produces *m/z* 257.1142 [M + Na]^+^ adduct ions in positive ionization mode. According to the parent ions and fragment ions, CP 39, CP 43 and CP 47 were identified by literature comparison as lobetyolinin, lobetyolin and lobetynol, respectively (Xie et al., 2023). Among them, lobetyolinin and lobetyolin were compared with reference substances. The possible cleavage pathway of lobetyolinin is shown in Figure 2A. The structural diagrams and fragmentation patterns of the three polyacetylenes are shown in Supplementary Figure S3.

From the results of Figure 2, the intensity of compound 35 (CP 35) in CT was much higher than that in CP and CM. CP 35 is an obvious difference in three origins and has the potential to become a marker compound to distinguish different origins of CR. CP 35 produced the parent ion of *m/z* 350.1971 in the positive ionization mode, and no other adduct ions that may be CP 35 were found in the same period. At the same time, no possible adduct ion of CP 35 was found in the negative ionization mode. It is speculated that CP 35 produces the adduct ion of [M]^+^ in the positive ionization mode, which may belong to the pyrrolidine alkaloid in CR, and the chemical formula is C19H28NO5^+^. The adduct ion of *m/z* 350.1971 [M]^+^ produced fragment ions of *m/z* 161.0599, 85,0761 and 83.0495 in the positive ionization mode. Compared with the ion data and relative peak time in the literature (Tang et al., 2022), CP 35 was confirmed to be codonopyrrolidium A. The possible cleavage mode is shown in Figure 2B, a total of 18 alkaloids have been identified, with 13 of them being pyrrolidine alkaloids like codonopyrrolidium A. These compounds have a positive ion on the nitrogen atom, resulting in the adduct ion of [M]^+^ in the positive ionization mode. The cleavage mode is similar to that of codonopyrrolidium A. The structure of 13 pyrrolidine alkaloids and the summary of the cracking rules are shown in Supplementary Figure S4.

Among the 7 lignans, CP16 (coniferoside), CP18 (syringin), and CP20 ((E)-isoconiferin) have the same parent nucleus structure and produce [M + Na]^+^ adduct ions in the positive ionization mode. The adduct ion produces fragment ions of *m/z* 203 and *m/z* 185 under the action of collision energy. The structure of the three compounds and the summary of the cracking rules are shown in Supplementary Figure S5.

### 3.3 Origins discrimination of CR

#### 3.3.1 Screening of differential metabolites and ionization modes

**FIGURE 3:**
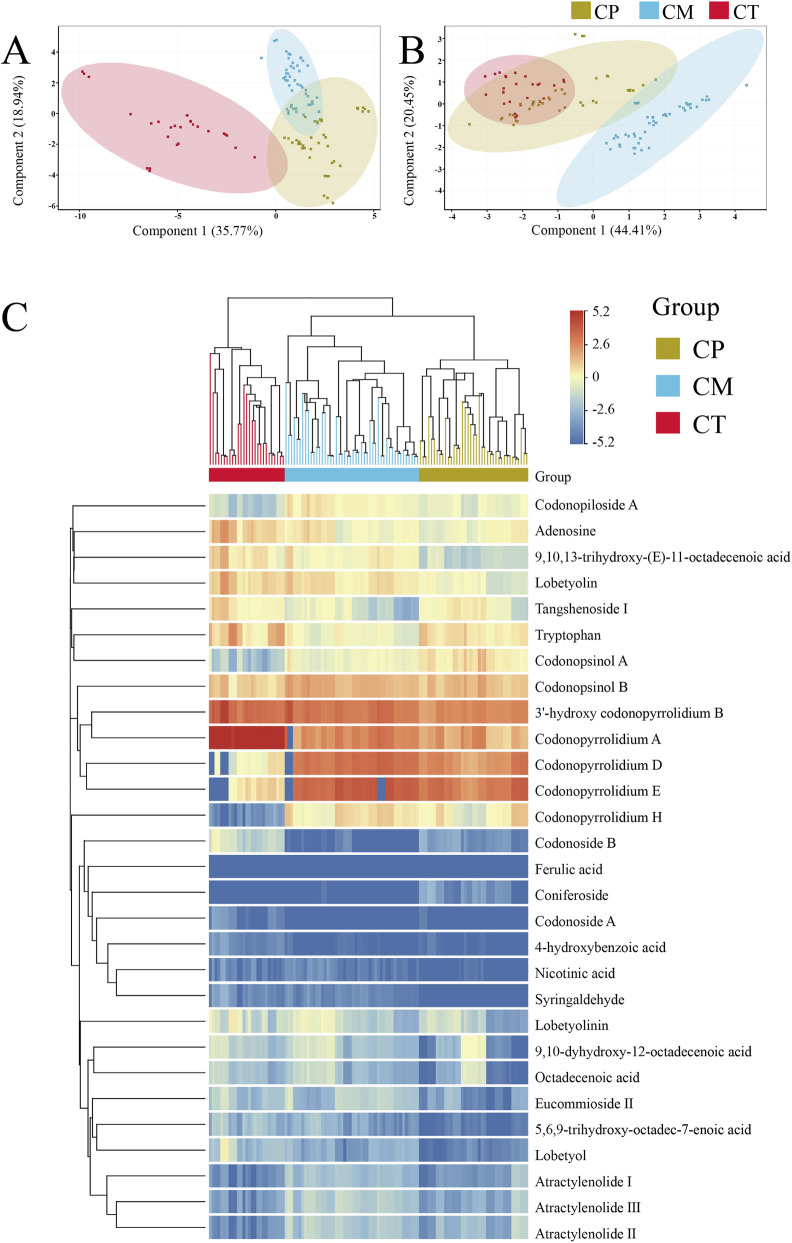
PCA clustering results in the positive ionization mode **(A)** and negative ionization mode **(B)**; heat map of 29 differential metabolites in the positive ionization mode **(C)**.

With CT as the central group, ANOVA was used to screen the differential metabolites with p < 0.05 and FC ≥2. Through analysis of variance, difference metabolites were obtained in positive ionization mode (Supplementary Table S2), as well as difference metabolites were obtained in negative ionization mode (Supplementary Table S3). The chemical structural formulas of 29 differential metabolites in positive ionization mode are shown in Supplementary Figure S6. Due to the analytical characteristics of non-targeted metabolomics, the use of more metabolic characteristics in chemometric analysis will produce more reliable results (Wang et al., 2023). In addition, from the metabolites list and different ionization mode screening results, organic acids in CR tend to be ionized in negative ionization mode, while alkaloids produce higher intensity in positive ionization mode. Taking these facts into account, we continued to use the differential metabolites in the positive and negative ionization modes for PCA analysis, then compared the separation results of different original CR samples in the two modes. The results are shown in Figures 3A,B. Obviously, the separation of CR samples was better in the PCA analysis results of positive ionization mode. Based on all the results, the differential metabolites obtained by positive ionization mode analysis are more suitable for the model establishment of different original CR.

The variation observed among metabolites can be attributed to their distinct biosynthetic pathways within the plant. This is exemplified by lobetyolin, a key active component and one of the identified differential metabolites in CR (Xu et al., 2024). Using pyruvate as the starting material, hexadecanoyl-acp is synthesized *via* a series of reactions, followed by the production of the saturated fatty acid palmitic acid catalyzed by the FATB enzyme. Palmitic acid is then converted into lobetyol through the unsaturated fatty acid pathway involving enzymes such as FAB2 and FAD2, ultimately derived from linoleic acid *via* dehydrogenation, oxidation, and hydroxylation reactions. Concurrently, the glucosyl group is produced from sucrose through enzymes including sucrose convertase, fructose kinase, and glucose phosphate mutase, resulting in UDP-glucose. Finally, a glycosyltransferase catalyzes the conjugation of the glucosyl group with lobetyol to form the final product, lobetyolin. In this process, differential expression of genes involved in the biosynthesis of lobetyolin can lead to variations in its content. However, current research on gene expression and biosynthesis in CR remains limited, with almost no comparative studies on these aspects among the three CR origins. Consequently, it is still challenging to explain the differences in metabolites among different CR origins from a deeper, more fundamental perspective.

**FIGURE 4:**
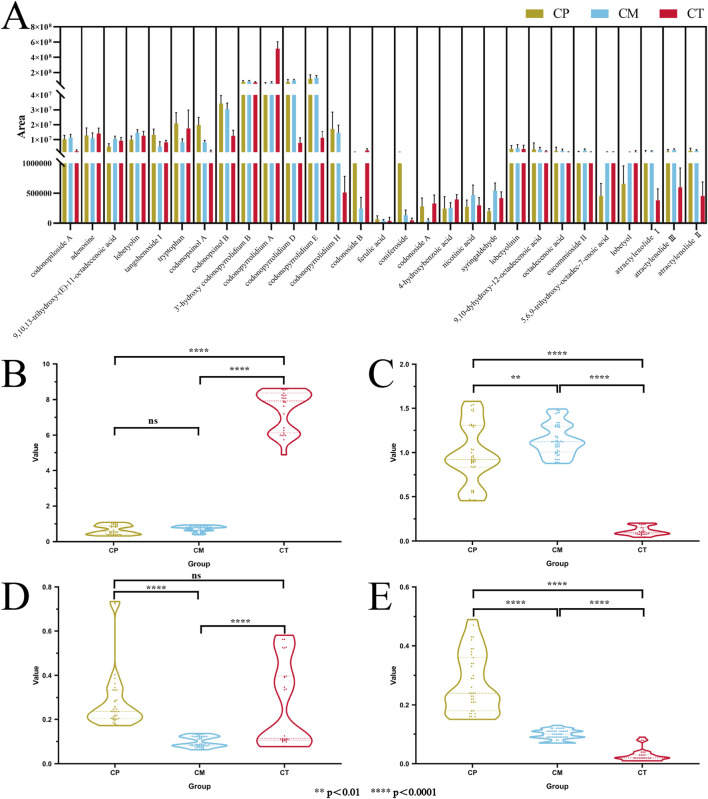
The histograms of the area of 29 differential metabolites in different sources **(A)**; violin plots of area ratio of codonopyrrolidium A **(B)**, codonopyrrolidium D **(C)**, tryptophan **(D)** and codonopsinol A **(E)** to 3′-hydroxy codonopyrrolidium B.

In order to better summarize and analyse the differences of differential metabolites in CP, CM and CT, and evaluate their identification ability, this study normalized the peak areas of differential metabolites in all samples and performed hierarchical cluster analysis (HCA) heat map analysis. The results are shown in Figure 3C, columns represent the CR samples, and each row represents the differential metabolites of the positive ionization mode. A color-coded scale grading from red to blue represents the area of differential metabolites from high to low. It can be clearly seen from the heat map results that the clustering effect between different origins is obvious. All the samples are divided into three categories, and the samples with the same origin are divided into one category. However, it was observed in the HCA that the response of most metabolites did not vary significantly after normalization. In order to better show the differences of metabolites in different groups, histograms were drawn with the mean and standard deviation (SD) of the area of differential metabolites in different origins of samples, as shown in Figure 4A. The histograms better show the differences in metabolites. It can be seen from the histograms the area of 4 pyrrolidine alkaloids, 3′-hydroxy codonopyrrolidium B, codonopyrrolidium A, codonopyrrolidium D and codonopyrrolidium E, is much higher than other metabolites. Among the 29 differential metabolites, 3′-hydroxy codonopyrrolidium B exhibited the smallest SD value, indicating that its content in each group of samples was similar and very stable. These metabolites with different contents in different origins may be derived from different origins, and the differences brought by the origins may directly distinguish the three original CR. Excellent examples already exist for the use of the peak area ratio method in the authentication of medicinal materials and their preparations. For instance, the *Japanese Pharmacopoeia* employs peak area ratios for quality evaluation of Curcuma. Furthermore, studies have established HPLC fingerprints for genuine (Peucedanum praeruptorum) and substitute species, where the peak area ratio of characteristic peaks (Chromatographic Peak 1/praeruptorin A and Chromatographic Peak 2/praeruptorin B) shows significant differences, enabling accurate differentiation (Chai et al., 2022). Similarly, in the analysis of *Rubia cordifolia* and its substitute, the peak area ratios involving specific characteristic peaks (e.g., Peak 1 to the peak of mollugin) exhibit marked variations, providing an effective means for discrimination (Chen et al., 2025). Therefore, this study attempted to calculate the area ratios of different metabolites and construct violin plots to differentiate the three origins based on the results.

To minimize the impact of concentration variations across samples, 3′-hydroxy codonopyrrolidium B with relatively stable content in all samples was selected as a reference. The area ratio of metabolites to 3'-hydroxy codonopyrrolidium B in each sample was calculated, and the distribution range of the ratio in different groups was described by violin plot. The analysis revealed that the results of codonopyrrolidium A and codonopyrrolidium D could well distinguish CT from the other two origins. As shown in Figures 4B,C, there is no overlap between the results of codonopyrrolidium A and codonopyrrolidium D in CT and the other two origins, and the differences were statistically significant. The area ratios of codonopyrrolidium A to 3′-hydroxy codonopyrrolidium B in CT were more than 4.5, while the area ratios in CP and CM were less than 1.2. The area ratios of codonopyrrolidium D to 3′-hydroxy codonopyrrolidium B in CT were below 0.21, while the area ratios in CP and CM were above 0.45. These results suggest that the area ratio of these two metabolites to 3′-hydroxy codonopyrrolidium B in a sample can be calculated to identify whether the sample belongs to CT according to the range of area ratio. The judgment results of the two compounds were mutually confirmed. If the results of the two area ratios were within the range of CT, it could be determined that the sample belonged to CT, otherwise it was other origin. The next objective is to screen for differential metabolites that could distinguish CP from CM. Among all the differential metabolites, only tryptophan and codonopsinol A could well distinguish CP and CM, as shown in Figures 4D,E. The area ratios of tryptophan to 3′-hydroxy codonopyrrolidium B in CP were above 0.17, and the ratios in CM were below 0.14. The area ratios of codonopsinol A to 3′-hydroxy codonopyrrolidium B in CP were above 0.15, and the ratios in CM were below 0.13. Significant differences were observed between the two groups for both ratios. The contents of tryptophan and codonopsinol A in different samples of CP were significantly different, while the contents in CM were relatively stable. The ratio of tryptophan and codonopsinol A to 3′-hydroxy codonopyrrolidium B in a sample was calculated. According to the interval of the ratio results, it can be judged whether the sample belongs to CP or CM.

The above experimental results show that by calculating the area ratio between metabolites, the origin of a sample can be quickly and accurately judged according to the ratio range. However, there will always be some errors in judging the sample base by one method. If multiple methods are used to judge and verify each other, the error of the results may be effectively reduced. In order to minimize errors, this study intends to construct another classification model to judge the origin of the sample together with the constructed area ratio method.

#### 3.3.2 Origin differentiation of CR

**FIGURE 5:**
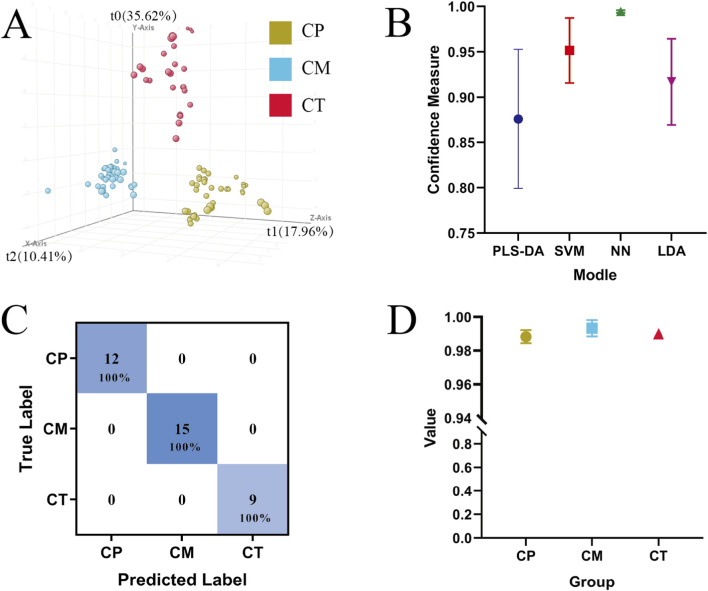
Sample clustering results by PLS-DA **(A)**; the confidence measures of four models **(B)**; validation algorithm outputs of the verification samples of new NN model **(C)**; the confidence measures of the verification samples of new NN model **(D)**.

In the early stage of the experiment, unsupervised PCA was used to observe the overall clustering trend and distribution trend between CR samples. As shown in Figure 3A, in the PCA model, the cumulative explained variance of the first two principal components is only 54.71%, and most of the differential metabolite information was not characterized. In the analysis results, the dispersion of CT samples is large, and there is a clear overlap between CP and CM, which will bring trouble to sample classification. Then developed a supervised Partial Least Squares Discriminant Analysis (PLS-DA) model to differentiate CR samples based on their origins. As illustrated in Figure 5A, the PLS-DA score plot displayed a distinct separation of CR samples from the three different origins in positive ionization mode. According to the validation algorithm outputs, the PLS-DA model demonstrates an overall accuracy of 100%, indicating that the prediction results of each sample align with the corresponding basis. However, the reliability and discriminant ability of the model cannot be proved only by the separation trend of the sample and overall accuracy. When the component is 3, excellent parameters (R2Y = 0.878) are obtained in the PLS-DA model, but the parameter (Q2Y = 0.245) becomes smaller, compared to the parameter (Q2Y = 0.710) when the component is 2. This reduction in Q2Y indicated that the PLS-DA model was overfitted. Despite being hallmark multivariate analysis techniques in metabolomics experiments, both PCA and PLS-DA proved to be clearly unsuitable for classifying the different origins of CR in this study (Worley et al., 2013).

In order to compare the classification ability of different analysis methods and obtain a more excellent model, this study also used Support Vector Machine (SVM), Neural Network (NN) and Linear Discriminant Analysis (LDA) methods to establish models to distinguish CP, CM and CT. The SVM model was constructed using a linear kernel with a cost parameter of 100 and a maximum of 100,000 iterations. The NN model consisted of 3 layers, trained over 100 iterations with a learning rate of 0.7 and a momentum of 0.3. The LDA model was also applied. All models were validated using the leave-one-out cross-validation method. According to the result of the validation algorithm outputs, the overall accuracy of these three models is 100% as good as PLS-DA. The confidence measure is introduced to compare the capabilities of different analysis methods. The confidence measure represents the strength of the prediction of belonging to a particular class. As shown in Figure 5B, the confidence measure of SVM, NN and LDA exceeded 0.90, and only the confidence measure of PLS-DA is less than 0.90. Among them, the NN model has the highest confidence measure, and the value is close to 1. NNs excel at identifying complex relationships among research variables and serve as an effective method for managing natural parameters (Cilla et al., 2012). This approach has been widely and successfully applied to various biological problems (Iglesias et al., 2014). For instance, one study demonstrated that a well-trained NN using electrical conductivity and the color coordinates a* and b* could effectively classify honey samples of different botanical origins (Anjos et al., 2015). Based on all the results, the NN model has better classification ability and is more suitable for classifying different original CR samples.

Both methods were analysed using the same set of differential metabolites. Ultimately, the ratio method utilized five specific metabolites: codonopyrrolidium A, codonopyrrolidium D, tryptophan, codonopsinol A, and 3′-hydroxy codonopyrrolidium B. Among these, the metabolites key for distinguishing CP and CM ranked 6th and 11th in weight within the NN model, indicating they are relatively important contributors. However, codonopyrrolidium A and codonopyrrolidium D, which were effective in differentiating CT in the ratio method, ranked lower in importance within the NN model (25th and 29th, respectively). This suggests that while these two metabolites can prominently distinguish CT in the straightforward ratio comparison, their overall contribution to the complex decision-making process of the NN model is not as high. This discrepancy likely stems from the fundamentally different principles of the two methods, leading them to rely on distinct sets of metabolites. Based on an analysis of the NN model’s structure, the contribution of each metabolite to the model was evaluated. The metabolites most critical for the classification decision were identified by ranking the sum of the absolute values of the input layer connection weights, with detailed weight data provided in Supplementary Table S5. These key metabolites showed a high degree of consistency with the results from the prior ANOVA and the VIP values of the PLS-DA model. For instance, syringaldehyde and codonoside B ranked first and second, respectively, in the NN model weight analysis, while they also ranked second and first, respectively, in the ANOVA results and the PLS-DA model VIP values. Furthermore, the top five compounds ranked by NN model weights were all within the top 10 in the ANOVA results, and their VIP values in the PLS-DA model were all greater than 1. This indicates that statistical methods based on different principles consistently point to the same set of core compounds, further confirming the reliability of these metabolites as biomarkers for species discrimination.

#### 3.3.3 Discriminant ability evaluation of NN model

To evaluate the identification and predictive capabilities of the NN model constructed with differential metabolites, 2/3 of each original CR sample (n = 78) were re-analyzed using the screened differential metabolites to create a new NN model (Wang et al., 2023). The output results of the verification algorithm (Figure 5C) show that the total accuracy of the NN model is still 100%, with the confidence measure of the model is greater than 0.99. This shows that the reconstructed model is as good as the original and has good effectiveness.

Ultimately, an external validation test was performed on the remaining one-third of the CR samples of each original (n = 36), and the original classification of each sample was performed. The results proved the availability of the newly established NN model, which are shown in Figure 5D. The accuracy of the verification experiment is 100%, and each sample falls on the corresponding origins. Moreover, the confidence measure of the prediction results of each original sample is above 0.98. These results further confirmed that the new NN model has strong practicability. Therefore, the NN model established by using the differential metabolites identified and screened through untargeted metabolomics based on UHPLC-Q/TOF-MS exhibits a strong capability to differentiate the origin of CR.

## 4 Conclusion

This study established a rapid, stable, and reliable UHPLC-Q/TOF-MS method, identifying a total of 56 metabolites in CR. Among these, 29 showed significant differences among the three origins of CR in positive ion mode. First, a simple and quick ratio method was developed to distinguish the three origins, calculating the response ratios of codonopyrrolidium A, codonopyrrolidium D, tryptophan, and codonopsinol A against 3′-hydroxy codonopyrrolidium B. The origin of CR samples was determined based on the range of these ratios. Subsequently, a NN classification model was established for the same purpose, achieving an accuracy rate of 100% with a precision close to 1. The ratio method is simple and efficient, while the NN model provides high accuracy. The classification results from both methods mutually validate each other, further enhancing the accuracy of CR classification. The findings of this study can provide guidance for the rational use and development of CR.

## Data Availability

The raw data supporting the conclusions of this article will be made available by the authors, without undue reservation.
