# Supplementary material for: UHPLC-Q/TOF-MS-based differential metabolite screening and origins classification of Codonopsis Radix
Source: Front Pharmacol. 2026 Feb 18;17:1751079. doi: 10.3389/fphar.2026.1751079 (PMC12957242; doi:10.3389/fphar.2026.1751079)
Supplement: Supplementary file 1 [file DataSheet1.pdf]

Supplementary Material

1 Supplementary Figures and Tables

1.1 Supplementary Tables

Supplementary Table 1. Information of all Codonopsis Radix samples.

No	Origin	Batch number	Habitat
1	*Codonopsis pilosula* (Franch.)Nannf	LQ20230901	Dingxi City, Gansu province
2		LQ20230902	
3		LQ20230903	
4		DS-001	Min County, Gansu Province
5		DS-002	
6		DS-003	
7		DS-004	
8		DS-005	
9		DS-007	Weiyuan County, Gansu Province
10		DS-008	
11		DS-009	Luzhou District, Shanxi Province
12		DS-010	
13		DS-006	Shouyang Town, Gansu Province
14	*Codonopsis tangshen* Oliv	SW230901	Wushan County, Chongqing Municipality
15		SW230902	
16		SW230903	
17		CDS-202403	
18		CDS-202406	
19		CDS-202401	Enshi City, Hubei Province
20		CDS-202402	
21		CDS-202404	
22		CDS-202405	
23	*Codonopsis pilosula* Nannf.var.*modesta* （Nannf.）L.T.Shen	20230901	Wen County, Gansu Province
24		20230902	
25		221001	
26		221002	
27		221003	
28		SHDS-001	
29		SHDS-002	
30		SHDS-003	
31		SHDS-004	
32		SHDS-005	
33		SHDS-006	
34		SHDS-007	
35		SHDS-008	

36	SHDS-009	
37	SHDS-010	
38	SHDS-202401	Longnan City, Gansu Province

Supplementary Table 2. Differential metabolites screened in positive ionization mode.

No	Metabolity	No	Metabolity
1	3'-hydroxy codonopyrrolidium B	16	codonopyrrolidium H
2	4-hydroxybenzoic acid	17	codonoside A
3	5,6,9-trihydroxy-octadec-7-enoic acid	18	codonoside B
4	9,10,13-trihydroxy-(E)-11-octadecenoic acid	19	coniferoside
5	9,10-dyhydroxy-12-octadecenoic acid	20	eucommioside II
6	codonopsinol A	21	ferulic acid
7	codonopsinol B	22	lobetyolinin
8	adenosine	23	lobetyolin
9	atractylenolide II	24	lobetyol
10	atractylenolide I	25	nicotinic acid
11	atractylenolide III	26	octadecenoic acid
12	codonopiloside A	27	syringaldehyde
13	codonopyrrolidium A	28	tangshenoside I
14	codonopyrrolidium D	29	tryptophan
15	codonopyrrolidium E		

Supplementary Table 3. Differential metabolites screened in negative ionization mode.

No	Metabolity
1	hexyl-β-D-glucopyranosyl- (1→ 2)-β-D-glucopyranoside
2	hexyl-β-D-glucopyranosyl- (1→ 6)-β-D-glucopyranoside
3	atractylenolide III
4	codonoside A
5	codonoside B
6	eucommioside II
7	lobetyolin
8	tangshenoside I
9	woodorien

Supplementary Table 4. Statistical details for the metabolite area ratios shown in Figure 4 (Mean ± SD).

Metabolite Area Ratio	CP	CM	CT	ANOVA *p*-value	Post-hoc Comparisons (Tukey's HSD)
codonopyrrolidium A/3'-hydroxy codonopyrrolidium B	0.66± 0.26	0.75± 0.15	7.44± 1.15	<0.0001	CP vs. CM: p = 0.7706<br>CP vs. CT: p ≤ 0.0001<br>CM vs. CT: p ≤ 0.0001
codonopyrrolidium D/3'-hydroxy codonopyrrolidium B	0.98± 0.34	1.14± 0.18	0.11± 0.05	<0.0001	CP vs. CM: p =0.0036<br>CP vs. CT: p ⩽0.0001<br>CM vs. CT: p ⩽0.0001
tryptophan/3'-hydroxy codonopyrrolidium B	0.28± 0.14	0.10± 0.02	0.26± 0.19	<0.0001	CP vs. CM: p ≤ 0.0001<br>CP vs. CT: p = 0.7261<br>CM vs. CT: p ≤ 0.0001
codonopsinol A/3'-hydroxy codonopyrrolidium B	0.27± 0.10	0.10± 0.02	0.03± 0.02	<0.0001	CP vs. CM: p ≤ 0.0001<br>CP vs. CT: p ≤ 0.0001<br>CM vs. CT: p ≤ 0.0001

Supplementary Table 5. The weights of 29 differential metabolites in the NN model.

Metabolite	Weight
syringaldehyde	7.45
codonoside B	7.234
atractylenolide I	6.277
9,10,13-trihydroxy-(E)-11-octadecenoic acid	5.898
codonoside A	5.818
codonopsinol A	5.744
5,6,9-trihydroxy-octadec-7-enoic acid	5.678
tangshenoside I	5.418
adenosine	5.335
codonopyrrolidium H	5.29
tryptophan	5.263
lobetyol	5.15
codonopyrrolidium E	5.046
coniferoside	5.039
9,10-dyhydroxy-12-octadecenoic acid	4.924
3'-hydroxy codonopyrrolidium B	4.772
codonopiloside A	4.679
atractylenolide II	4.564
eucommioside II	4.544
4-hydroxybenzoic acid	4.463
ferulic acid	4.356
atractylenolide III	4.31

codonopsinol B	4.291
lobetyolinin	4.197
codonopyrrolidium A	4.039
lobetyolin	3.865
nicotinic acid	3.768
octadecenoic acid	3.228
codonopyrrolidium D	2.885

1.2 Supplementary Figures

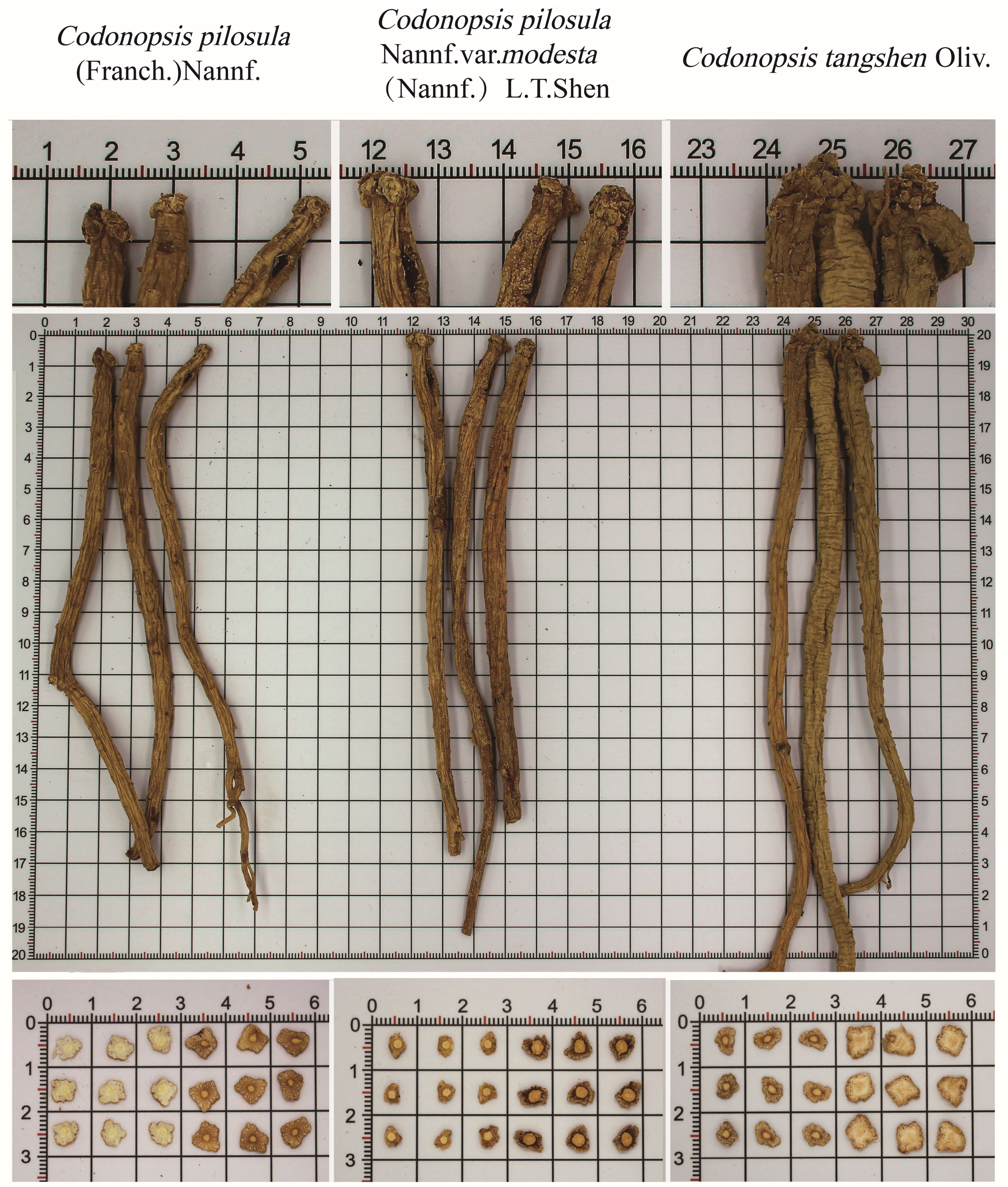

Supplementary Figure 1. Appearance characters and herbal slices of CP, CM and CT.

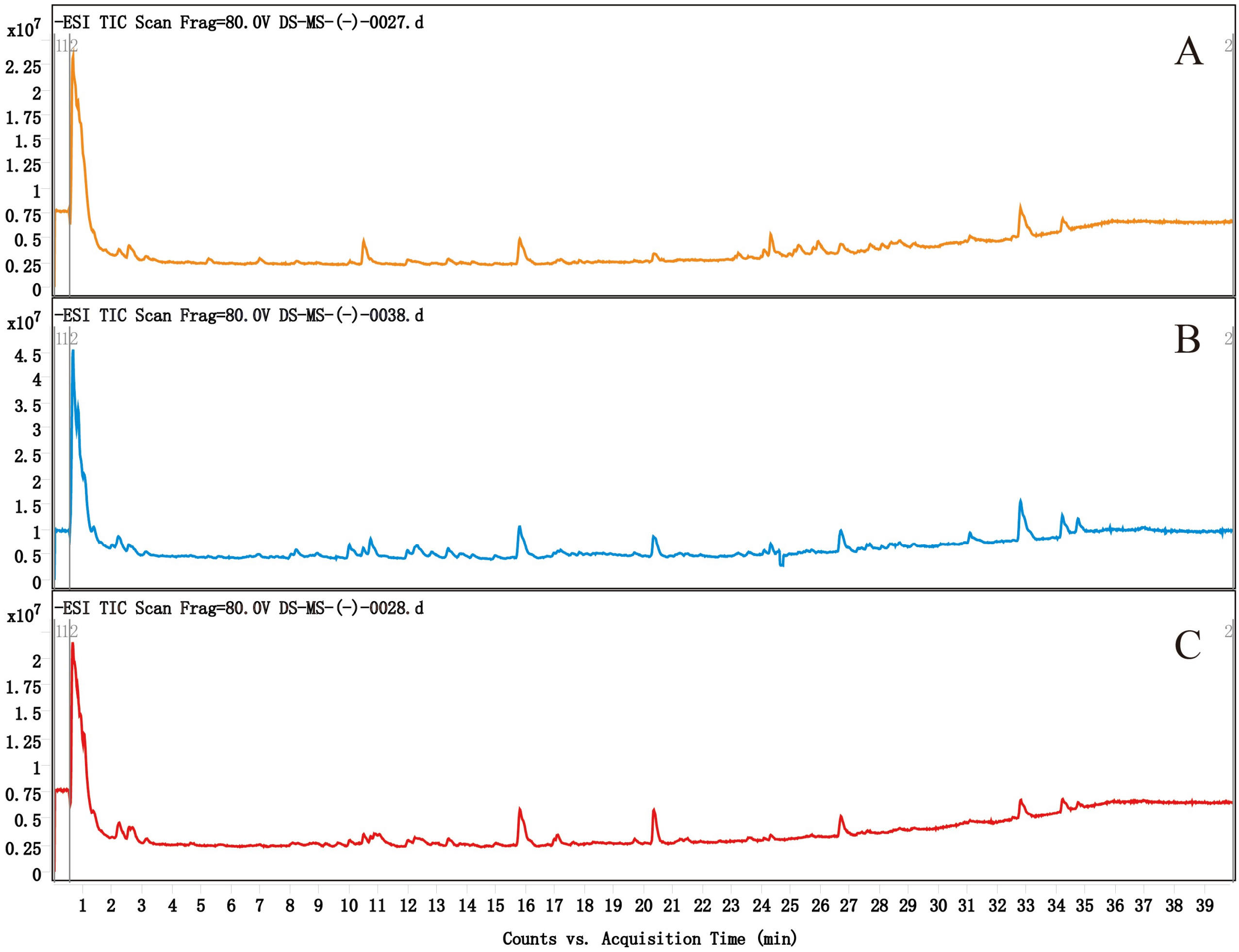

Supplementary Figure 2. TIC of CP (A), CM (B) and CT (C) in ESI-.

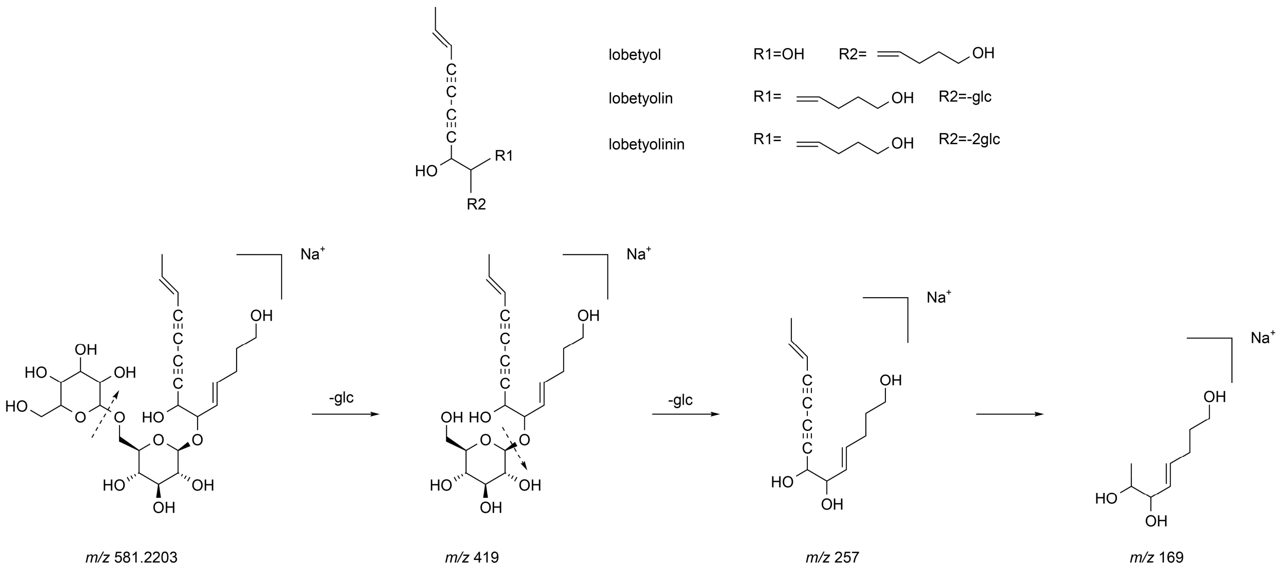

Supplementary Figure 3. The structural diagrams and fragmentation patterns of the three polyacetylenes.

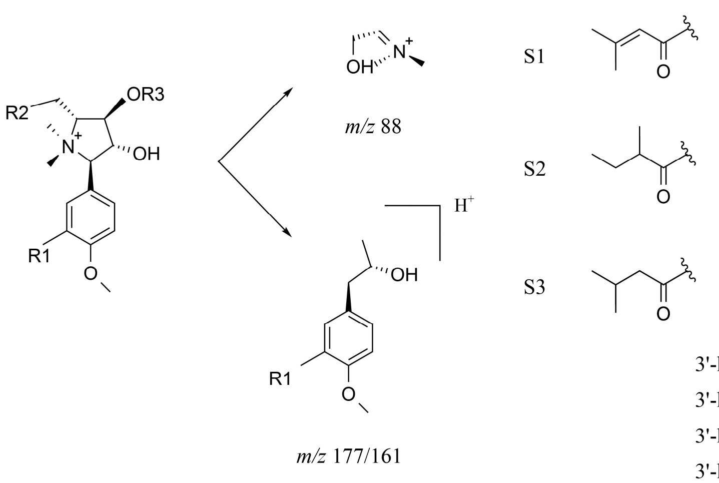

codonopyrrolidium A	R1=H R2=H R3=S1
codonopyrrolidium B	R1=H R2=H R3=H
codonopyrrolidium C	R1=H R2=S1 R3=H
codonopyrrolidium D	R1=H R2=S2 R3=H
codonopyrrolidium E	R1=H R2=S3 R3=H
codonopyrrolidium F	R1=H R2=H R3=S2
codonopyrrolidium G	R1=H R2=H R3=S3
codonopyrrolidium H	R1=OH R2=S3 R3=H
codonopyrrolidium I	R1=OH R2=H R3=S1
3'-hydroxy codonopyrrolidium B	R1=OH R2=H R3=H
3'-hydroxy codonopyrrolidium D	R1=OH R2=S2 R3=H
3'-hydroxy codonopyrrolidium F	R1=OH R2=H R3=S2
3'-hydroxy codonopyrrolidium G	R1=OH R2=H R3=S3

Supplementary Figure 4. The structure of 13 pyrrolidine alkaloids and the summary of the cracking rules.

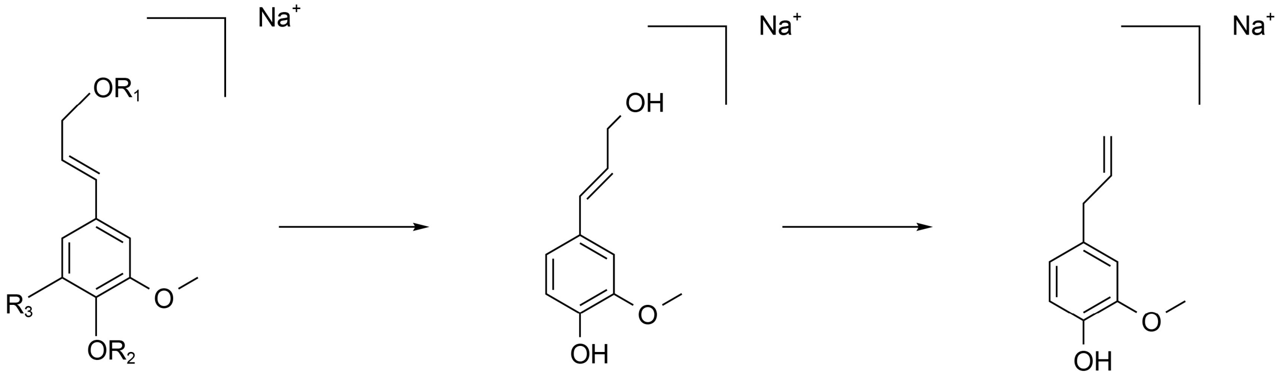

m/z 203   *m/z* 185

coniferoside	R_1=H R_2=-glc R_3=H
(E)-isoconiferin	R_1=-glc R_2=H R_3=H
syringin	R_1=H R_2=-glc R_3=OCH_3

Supplementary Figure 5. The structure of the three compounds and the summary of the cracking rules.

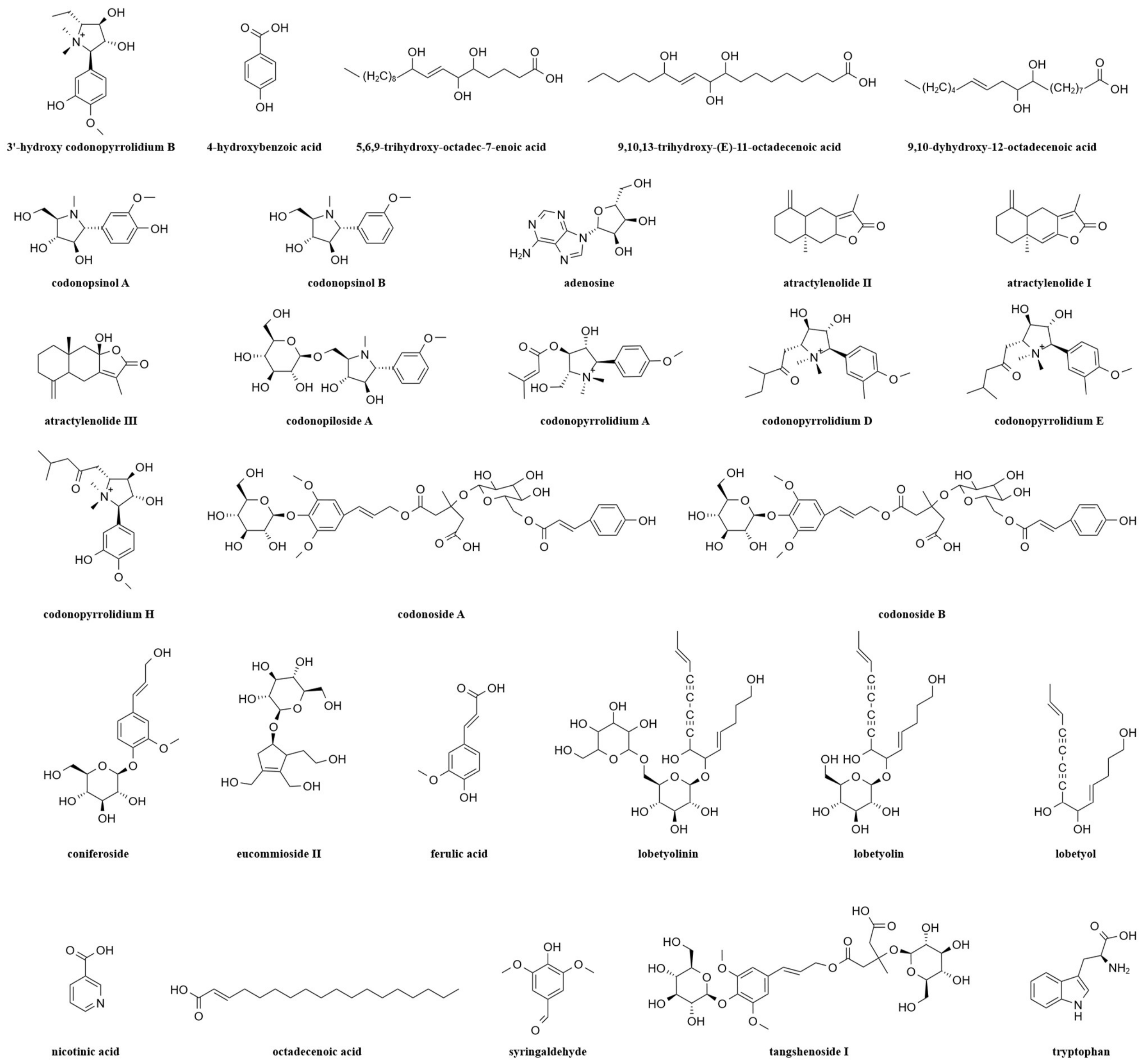

Supplementary Figure 6. The chemical structural formulas of 29 differential metabolites.
